# Subjective Assessment of Sleep in Infantile Autism: A Comparative Study

**DOI:** 10.3390/bs9020012

**Published:** 2019-01-24

**Authors:** Maydelin Alfonso-Alfonso, Lilia María Morales-Chacón, Justa Elizabeth González-Naranjo

**Affiliations:** Neurophysiology Department, International Center for Neurological Restoration, 25th Ave, No 15805, 11300 Havana, Cuba; lily@neuro.ciren.cu (L.M.M.-C.); egonzalez@neuro.ciren.cu (J.E.G.-N.)

**Keywords:** Subjective sleep assessment, Autism spectrum disorder, REM sleep, NREM sleep

## Abstract

Sleep disturbances are very common in children with autism; it is for this reason that instruments that facilitate their evaluation are necessary. Objectives: Perform sleep assessment from a subjective perspective in a group of children with primary autism and compare them with a control group, using the Sleep Habits in Children Survey (CSHQ), with the purpose of determining sleep disturbances according to the subscales used. Method: A prospective cross-sectional study was conducted in a group of 21 patients with primary autism. For the evaluation of sleep disturbances, we chose the CSHQ survey. The differences between the independent groups were calculated by applying a Mann–Whitney U test. Results: In the group of children with autism, higher values of the total scale were observed in comparison with the control group (*p* = 0.00) which It is congruent with a large sleep dysfunction. Significant differences were observed for all subscales (*p* = 0.00), with the exception of the subscale number 7. Conclusions: A high presence of sleep disturbances was observed in children with primary autism, with the exception of sleep breathing disorders, which did not show significant differences between the groups.

## 1. Introduction

Autism is a neurological development disorder characterized by a deficit in three main domains: social interaction, language development and communication. In these children, sleep disturbances are very common [1,2].

Sleeping is one of the necessary functions for all people. Sleep in children follows a process of development that begins from the fetal stage, and shows successive changes in the course of childhood. The configurations of the no rapid eye movement (NREM) sleep and rapid eye movement (REM) sleep cycles and the durations of their stages are also very important from the first months of life since they intervene in the whole process of maturation of the nervous system [1,2].

With a prevalence of 20–50 cases per 10,000 inhabitants, autism is more frequent in males (3:1) and generally, the clinical manifestations begin after the first year of life [3]. Autistic children usually have difficulty sleeping and between 33% and 44% of children with autism suffer from a sleep disorder [4,5] with insomnia of onset and maintenance predominating. In addition, it has been described that insomnia in autistic children alters the parents’ sleep and adds great stress to the lives of their families [6]. The hypotheses about the intrinsic causes of insomnia in these children include the organizational and maturational differences of brain waves, genetic constitution, abnormal production of melatonin and sensory deregulation [7].

Sleep disturbances and daytime sleepiness that occurs because of these, have a negative impact on the behavior of these children, which often manifests as hyperactivity, lack of attention and aggressiveness [8,9]. It has been suggested that the neurobiological causes of sleep disorders in autism are abnormalities in gamma amino butyric acid (GABA), a neurotransmitter involved in the generation and maintenance of a regular cycle of wakefulness and sleep. Genetic abnormalities have also been described which involve the clock genes [10,11,12].

On the other hand, it has been reported that melatonin in blood and in urine decreases in autism, which leads to alterations in the circadian rhythm [13,14]. Low melatonin and clock gene anomalies in people with autism seem to be involved in social and circadian problems [15]. Melatonin is a hormone synthesized in the pineal gland, presenting a pattern of circadian concentration with low levels of concentration during the day and high levels during the night, contributing to the regulation of circadian rhythms [16]. In a study conducted in a small group of patients, plasma melatonin profiles are comparable to those reported in the literature for children with a typical development, in these cases the onset of sleep occurred when plasma melatonin levels increased [17].

Sleep disturbances in individuals with autism can be attributed to numerous factors among which are environmental, biological, psychological and social factors [18]. These were the reasons that motivated us to carry out this study, considering that it is very important to have an instrument that provides us with data not only about the night’s sleep, but also about the diurnal habits and social interaction of these patients. With these data, we could complete the sleep assessment of autistic children that we receive frequently in the neurophysiology laboratory.

In a previous study to this investigation, we conducted a review of the different questionnaires proposed in the literature to find an instrument that we could use in our laboratory [19,20,21,22,23]. We chose (CSHQ) since it is a retrospective questionnaire and evaluates sleep habits in children between 2 and 10 years of age, collecting data from the previous week, which reduces the risk of error by subjectivity. The questionnaire is an abbreviated version of 33 elements that includes the symptoms of the most common sleep problems according to the international classification of sleep disorders; these symptoms were grouped into eight subscales [23]. This questionnaire was validated in English and to facilitate the understanding of the parents, we validated the questionnaire in Spanish and adapted it to the sociocultural conditions of Cuban families.

Taking into account the benefits of this instrument, we focus our attention on childhood autism. We set out to carry out a sleep evaluation from a subjective perspective in a group of children with primary autism and compare these results with a control group, using the Sleep Habits Survey in Children (CSHQ). The main objective of this research is to determine the sleep disturbances observed according to the subscales applied. Frequent sleep disturbances were observed in autistic children, which is consistent with what is described in the literature.

## 2. Materials and Methods

A prospective cross-sectional study was conducted in the period between November 2016 and February 2017. Previously in 2015, a validation work of the applied questionnaire was carried out, in which the observed results showed that this instrument had the adequate psychometric properties to evaluate the sleep habits of Cuban children in the age range of interest, being a highly reliable instrument (this article is in the process of revision for its current publication in the Cuban pediatric journal).

Healthy controls were recruited in schools and children’s centers. We selected the group of autistic children who were seen in neurology clinics in other medical centers, and were subsequently seen in our laboratory with a confirmed diagnosis of primary autism [24]. Both groups were selected to obtain a sample as homogeneous as possible.

The sample was matched in age with an average age of 5.23 years (mean/5.23, SD/1.99) but not in sex, obtaining a sample of 21 children per group. We selected for each group: 5 children of 3 years, 4 children of 4 years, 4 children of 5 years, 2 children of 6 years, 3 children of 7 years, 2 children of 8 years, 1 children of 10 years (Table 1). The lack of response of 20% of the questionnaire as well as those parents who refused to participate in the study were also considered as exclusion criteria.

The group of two-year-old patients was excluded (because they did not have a definitive diagnosis of autism) and the children who were receiving some psychostimulant medication, anticonvulsants or antihistamines, since they are medicines that can affect the normal sleep architecture [25]. For the statistical analysis, Statistic 8 was used (Statistic 8.0.360 Copyright Stat Soft, Inc., Tulsa, OK, USA, 1984–2011). The differences between independent groups were calculated applying a Mann–Whitney U test. The values of *p* were considered significant below 0.05.

In Table 2 we show the questionnaire evaluation method, with the items that each subscale evaluates, which in turn are in correspondence with sleep disturbances [23]. Sleep behavior is classified on a scale of 3 points, generally 3 means 5 to 7 times a week, 2 means between 2 to 4 times a week and 1 (rarely) means between 0 to 1 times per week. The score of the items was inverted (1, 2, 3, 11, 26) so that the highest score corresponds to the most disturbed sleep.

All procedures followed the 2013 Declaration of Helsinki rules for research on human subjects, and the study was approved by the scientific and ethical committee (CIREN 63/2015) of the International Center for Neurological Restoration (CIREN). (https://www.wma.net/policies-post/wma-declaration-of-helsinki-ethical-principles-for-medical-research-involving-human-subjects/).

## 3. Results

The results observed by the application of the questionnaire showed that the autistic children presented the highest values when the survey was analyzed globally, this total result showed an average of (48.00), while the control group presented an average of (36.47). (*p* = 0.000) (Graph 1) 3.1.1 Significant differences were also found for all the subscales of the questionnaire with *p* = 0.00. Only subscale 7 did not show significant differences between groups (*p* = 0.61). This subscale evaluates the presence of respiratory disorders related to sleep, although it is a pathology that usually occurs in relation to the comorbidities suffered by autistic children, as in the case of obesity, which in these patients was not observed (Table 3).

**Figure 1:**
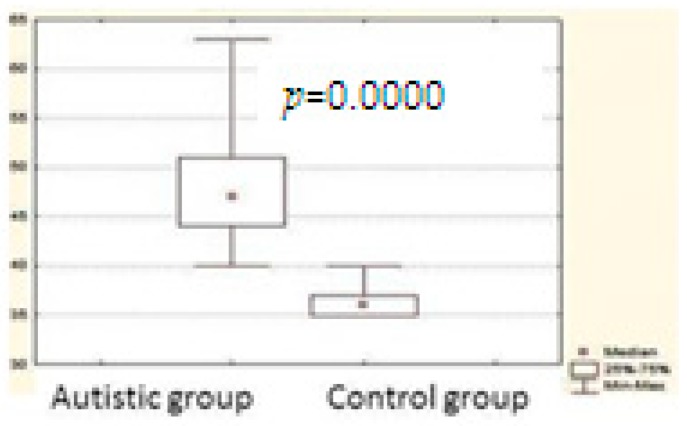
Differences between groups for the full scale. Comparison between cases of Autistic group (Group 1) and control group (Group 2). (Mean/Standard deviation).

The subscale that assesses the presence of parasomnias is also significantly affected. This is a sleep disorder that although it is not the most described in the literature, in our study it has been found as an element related to childhood autism in all cases, compared to healthy controls the difference was significant (*p* = 0.0000) (In Figure 1). We did not find significant differences between the ages of the group of autistic children and the control group. Related to sex, we observed the typical prevalence of male sex described in the literature in the group of autistic children (76%). Regarding the level of education of parents in both groups, the highest percentage prevailed at the level of higher education (67% of the control group and 62% of the autistic group).

## 4. Discussion

The differences observed between the groups of children with autism and healthy controls showed that sleep disturbances are highly prevalent in the group of children with primary autism in relation to the control group, which is in congruence with the literature. Sleep problems in autistic people are related to complex interactions between biological, genetic, psychological and environmental factors [18].

The autistic group presented significant differences for all the subscales evaluated by the applied questionnaire. This is consistent with alterations in the onset of sleep, duration of sleep, anxiety before sleep, night awakenings, parasomnias, as well as a high level of daytime sleepiness, which has a very negative effect on performance during the day, the learning and the quality of life of these children and their families.

In these children, clinical features and psychiatric comorbidities, such as pathological anxiety and depression, predispose to sleep problems. In fact, the somatic and cognitive hyperarousal states resulting from rumination and negative thoughts prevent the onset of sleep [24,25]. Other causes that predispose to sleep problems in children’s autism are inadequate sleep hygiene.

Conversely, patients with this diagnosis who show hypersensitivity to tastes or textures may feel more anxious at bedtime, for example, when brushing their teeth, which cause difficulties in falling asleep [26]. It can also be observed a negative influence on the adequate sleep process, associated diseases such as epilepsy, the use of medications such as antidepressants or psychostimulants [27], the presence of specific symptoms related to autism such as hyperactivity, obsessiveness, anxiety and stereotypes [28,29].

Autistic individuals have difficulty breaking routines, often falling asleep in the arms of their parents and this is a risk of not being able to go back to sleep alone after a spontaneous awakening.

Similarly, slight changes in the bedtime routine will lead to the onset of sleep later [30,31]. The alterations of the circadian rhythm in autistic children are related to a deficit of socialization. In fact, circadian synchronization is closely related to social interaction and the natural light-dark cycle [32]. In another report [33], it is suggested that the levels observed during the night of urinary excretion in these patients seem to be correlated with slow-wave sleep and inversely correlated with stage 2 of NREM sleep and daytime sleepiness.

With respect to clock gene abnormalities, studies report mutations in the NLGN/NRXN/SHANK3 complex (neuroligin/neurexin/synaptic scaffolding protein 3) in autistic individuals that produce sleep–wake disturbances [15].

Recent reports related to the high incidence of insomnia in these patients postulate that the theory of cognitive arousal (thinking and worrying while trying to fall asleep) prevents the initiation of the sleep process. One study showed greater activation of the sympathetic nervous system in patients with insomnia compared to good sleepers [34,35,36]. Often, insomnia is the result of an inappropriate association with sleep [37]: Falling asleep is associated with a form of stimulation (rocking chair, TV), object (bottle) or environments (room lit, parents in the room or falling asleep the parents’ bed). Its absence conditions the difficulties and its restoration facilitates sleep. Often we can see awakenings during the night, night fears or anxiety to sleep alone. It is considered a disorder if: the associations are very problematic and demanding; sleep onset is significantly delayed or sleep is interrupted in the absence of those conditions, and frequently requires the intervention of parents.

Difficulties with sleep onset, sleep maintenance and co-sleeping are most commonly found [38]. At bedtime, children with autism spectrum disorder are less likely to be sleepy, are likely to be noncompliant, may follow problematic bedtime routines, and may have reduced night sleep [39]. Other sleep problems include extended periods of night waking, often accompanied by behaviors that disturb the rest of the family (e.g., laughing, screaming, playing [40]; irregular sleep/wake patterns with variability in sleep onset and waking [41]; early morning waking [42]; and reduced sleep efficiency [43] compared with typically developing peers. Using both actigraphy and a sleep questionnaire to compare sleep in autism spectrum disorder and typically developing children aged 4 to 10 years, concluded that in autism spectrum disorder sleep problems were likely to be associated with the disorder; that is, these children had insomnia due to pervasive developmental disorder [38].

According to sleep diary studies, nap frequency may be lower in young children with autism, though reports can be contradictory and are likely related to age variation in the samples, the use of mixed disability groups for comparison, sample size, and the time period over which data are collected. Children with autism aged 4 years or younger slept less in a 24-h period than typically developing children, despite napping [41], and young children with autism spectrum disorder (<6 years) napped less frequently and for a shorter period than children with mixed disabilities or typically developing children [44]. However, when children with autism (mean age 7.2 years) were compared to similar age children typically developing. Young children with autism spectrum disorder may take shorter naps or fewer naps, thus sleeping less over a 24-h period than other children. This is important in terms of determining the origin of sleep problems and whether the child is getting sufficient sleep, as it is the total sleep each 24 h that determines whether the child’s sleep is sufficient.

On average, children with autism spectrum disorder show a reduction in total night sleep compared with typically developing children or age- and IQ-matched controls, and this difference is often significant [39]. However, not all studies report significantly reduced night sleep time in comparison to other children [41].

Besides the more common settling and night waking difficulties, parents of children with autism spectrum disorder also report a number of other sleep difficulties. Recent research using sleep questionnaires containing subscales measuring behaviors associated with Parasomnias suggest that these sleep phenomena may be relatively common in children with autism spectrum [45] disorder. Parasomnias may also relate to parent reports that some of these children wake screaming, possibly suggesting a susceptibility to night terrors.

Therefore, we can conclude that there is a high prevalence of sleep problems in the group of children with primary autism compared to the control group, with the exception of sleep-related respiratory disorders (subscale 7).

The prevalence of obstructive sleep apnea (OSA) in children has been estimated between 1% and 4%, but this prevalence may currently be higher. This disease can occur at any age, from the neonatal period to adolescence, but it is more common in pre-school age being associated with adenotonsillar hypertrophy and in adolescents associated with obesity.

Complications are frequent and may be severe. Cognitive and behavioral complications are common and may include developmental delay, poor school performance, attention deficit hyperactivity disorder, inattention and impairment in concentration, and aggressive behavior. Excessive daytime sleepiness may be present [37]. That why it is very important to be clarify this problem in children with autism. 

The use of questionnaires is insufficient in order to diagnoses this disease, but some authors propose a new questionnaire using the machine learning models; they can stratify OSA with higher accuracy [46].

In the other subscales we found affectations that show significant differences between the groups of children with autism and healthy controls. These subscales evaluate the most common sleep disorders according to the International Academy of Sleep Disorders [37]. The described alterations are closely related to complex interactions between biological, genetic, psychological and environmental factors, and are involved in the sleep problems of individuals with autism spectrum disorders, which is in accordance with what is reported in the updated literature.

The evaluation from a subjective perspective is an alternative to the realization of polysomnography (PSG). Questionnaires for parents and caregivers can be used in these patients, through a structured survey, evaluating different parameters of sleep quality and behavior during sleep. In future studies, we propose to complete this evaluation with some objective sleep studies that allow us to investigate in greater depth sleep disorders in children’s autism and make new comparisons with patients with sleep disorders and other comorbidities (for example, epilepsy).

## 5. Limitation of the Study

We believe that we should increase our sample to relate the sleep disorders observed in the survey with the severity of autism and socioeconomic status. Neither do we mention if we do some kind of intervention to improve sleep behavior in these children.

## Figures and Tables

**Table 1:** Sociodemographic characteristics of the sample.

	N	Age Years u(SD)	Sex		Schooling of Parents		
			F	M	Low	Medium	Higher
Autistic group	21	5.23 (1.99)	5 (23%)	16 (76%)	2 (9%)	6 (29%)	13 (62%)
Control	21	5.23 (1.99)	12 (57%)	9 (42%)	0	7 (33%)	14 (67%)

**Table 2:** Evaluation of sleep habits survey in children (CSHQ).

Subscales	Items
1. Bedtime resistance	1,3,4,5,6,8
2. Sleep onset	2
3. Sleep duration	9,10,11
4. Anxiety prior to sleep	5,7,8,21
5. Awakenings at night	16,24,25
6. Parasomnias	12,13,14,15,17,22,23
7. Respiratory sleep disorders	18,19,20
8. Daytime drowsiness	26,27,28,29,30,31,32,33

**Table 3:** Differences between groups for the full scale and subscales.

	Group 1	Group 2	*p*-Value
Total scale	671.0000	232.0000	0.000000 *
Subscale 1	610.5000	292.5000	0.000063 *
Subscale 2	546.0000	357.0000	0.017444 *
Subscale 3	556.5000	346.5000	0.008258 *
Subscale 4	581.0000	322.0000	0.001123 *
Subscale 5	639.0000	264.0000	0.000002 *
Subscale 6	639.0000	264.0000	0.000002 *
Subscale 7	471.5000	431.5000	0.614884
Subscale 8	585.0000	318.0000	0.000784 *

Comparison between cases of Autistic group (Group 1) and control group (Group 2). (Mean) * *p* ≤ 0.05.
